# Effect of Genetic Variants in Two Chemokine Decoy Receptor Genes, *DARC* and *CCBP2*, on Metastatic Potential of Breast Cancer

**DOI:** 10.1371/journal.pone.0078901

**Published:** 2013-11-15

**Authors:** Chen Yang, Ke-Da Yu, Wen-Huan Xu, Ao-Xiang Chen, Lei Fan, Zhou-Luo Ou, Zhi-Ming Shao

**Affiliations:** Department of Breast Surgery, Cancer Center and Cancer Institute, Shanghai Medical College, Fudan University, Shanghai, China; University of South Alabama, United States of America

## Abstract

The inhibitory effect of two chemokine decoy receptors (CDRs), DARC and D6, on breast cancer metastasis is mainly due to their ability to sequester pro-malignant chemokines. We hypothesized that genetic variants in the *DARC* and *CCBP2* (encoding D6) genes may be associated with breast cancer progression. In the present study, we evaluated the genetic contributions of *DARC* and *CCBP2* to metastatic potential, indicated by lymph node metastasis (LNM). Ten single-nucleotide polymorphisms (SNPs) (potentially functional SNPs and block-based tagging SNPs) in *DARC* and *CCBP2* were genotyped in 785 breast cancer patients who had negative lymph nodes and 678 patients with positive lymph nodes. Two non-synonymous SNPs, rs12075 (G42D) in *DARC* and rs2228468 (S373Y) in *CCBP2*, were observed to be associated with LNM in univariate analysis and remained significant after adjustment for conventional clinical risk factors, with odds ratios (ORs) of 0.54 (95% confidence interval [CI], 0.37 to 0.79) and 0.78 (95% CI, 0.62 to 0.98), respectively. Additional functional experiments revealed that both of these significant SNPs could affect metastasis of breast cancer in xenograft models by differentially altering the chemokine sequestration ability of their corresponding proteins. Furthermore, heterozygous GD genotype of G42D on human erythrocytes had a significantly stronger chemokine sequestration ability than homozygous GG of G42D *ex vivo*. Our data suggest that the genetic variants in the CDR genes are probably associated with the varied metastatic potential of breast cancer. The underlying mechanism, though it needs to be further investigated, may be that CDR variants could affect the chemokine sequestration ability of CDR proteins.

## Introduction

The major cause of treatment failure and mortality from breast cancer is metastasis to distant organs [1]. Because breast cancer is a highly heterogeneous disease, it is a major challenge to predict the outcome of an individual patient [2]. Intensive research is currently underway to identify the genetic determinants that have a high diagnostic power for prognosis prediction, which would help identify patients who have a high risk of metastasis and to tailor therapeutic measures.

Chemokine and chemokine receptors have been highlighted for their vital roles in breast cancer progression and metastasis, involving proliferation, invasion, migration, senescence, angiogenesis, and regulation of host immune response [3], [4]. It is evident that CCL2 (MCP-1), CCL5 (RANTES), and CXCL8 (IL-8) have pro-malignant activity that is associated with breast cancer progression and more advanced disease [5]–[8]. Furthermore, functional single-nucleotide polymorphisms (SNPs) located in the promoter region of *CCL2* and *CXCL8*, and thus regulating transcription of these chemokines, have been associated with clinical breast cancer outcome [9]–[11].

Chemokines are not only mediated by transcriptional activity but also by posttranslational regulation. A chemokine decoy receptor (CDR), also called an atypical chemokine binder, is a new subgroup of chemokine receptors incapable of transmitting their signals through the classic G-protein-mediated pathways. They act as scavengers by efficiently internalizing their cognate chemokine ligands. The CDR group consists of at least three members: Duffy antigen receptor for chemokine (DARC), D6 (coded by *CCBP2*) and ChemoCentryx chemokine receptor (CCX-CKR) [12]–[14]. Previous research in our laboratory has demonstrated that DARC, D6, and CCX-CKR play inhibitory roles in breast cancer growth and metastasis, mainly by sequestration of the pro-malignant chemokines [15]–[17]. There are some potentially functional non-synonymous SNP in *DARC* and *CCBP2* but no non-synonymous ones were found in *CCX-CKR*, therefore we did not investigate the *CCX-CKR* variation in present study.

Lymphatic and hematogenous dissemination are two common ways for breast cancer cells to spread. DARC is widely expressed on erythrocytes and vascular endothelial cells [18] while D6 is mainly expressed on lymphatic endothelial cells [19]. DARC and D6 present on blood and lymphatic vessels and on erythrocytes in the circulation serve as a systemic barrier to metastasis. Given the broad distribution of CDRs within the body, their inhibitory effects on cancer progression and metastasis, and the potential influence of genetic variants on gene expression and protein activity, we hypothesized that breast cancer patients carrying certain CDR genotypes may be more susceptible to tumor spread. To test our hypothesis, we investigated the relationship between lymph node metastasis (LNM) and ten genetic variations in *DARC* and *CCBP2* in a cohort of patients with primary breast cancer. The biological mechanism was subsequently examined.

## Materials and Methods

### Ethics statement

All participants provided their written consent to participate in this study. This study was approved by the Science and Ethics Committee of the Shanghai Cancer Center and conforms to the principles outlined in the Declaration of Helsinki (IRB number: 050432-4-10087A).

All animal work was conducted in accordance with the National Institutes of Health ‘Guide for the Care and Use of Laboratory Animals’. The study protocol was approved by the Shanghai Medical Experimental Animal Care Committee.

### Study subjects

The candidates for this study were from consecutive female patients at the Shanghai Cancer Hospital (between Jul.2006 and Dec.2008) with pathologically confirmed operable primary invasive breast cancer. Subjects were identified as genetically unrelated Han Chinese from the Shanghai City and its surrounding regions. All patients underwent mastectomy or lumpectomy plus level I/II axillary lymph node dissection or sentinel node biopsy. Patient characteristics and tumor features were extracted from clinical documents. All data were eventually integrated into a computerized database established by our department, as described elsewhere [20]. The patients were excluded from this study if they had received neoadjuvant treatment or had bilateral breast cancer, ductal carcinoma *in situ*, or any history of other cancers. The histological type of the primary tumors was evaluated according to the WHO classification, and the Ellis&Elston system was used for histologic grading. The estrogen receptor (ER), progesterone receptor (PR), and human epidermal growth factor receptor-2 (HER2) statuses were determined by immunohistochemical staining, as previously described [21]. Routine pathologic examination of the lymph nodes was done with hematoxylin and eosin (H&E) staining; patients who had only micro-metastasis but no evidence of macro-metastasis were also excluded from the study. Finally, there were a total of 1,463 cases included, 785 with lymph node negative disease and 678 with lymph node positive disease.

### Cell lines

The human breast cancer cell line MDA-MB-231 was obtained from the American Type Culture Collection (ATCC). Liquid nitrogen cell stocks were made upon receipt and stored until the start of each study. Morphology and doubling times were also recorded regularly to ensure maintenance of the phenotype. Cells were used for no more than 6 months after being thawed (most experiments were finished within approximately 4 months). Thus, all of the cell lines have been tested and authenticated by ATCC and maintained in our laboratory for fewer than 6 months, during which all experiments were conducted. Cells were routinely cultured in Leibovitz's L-15 medium supplemented with 10% fetal bovine serum at 37° in a humidified 5% CO_2_ atmosphere.

### Plasmid constructs

*DARC* and *D6* expression vectors were constructed using the pcDNA3.1^(+)^ plasmid (Invitrogen, USA). The full-length human cDNA for *DARC* and *CCBP2* were amplified using the primers listed in **Table S1**. The fragment of *DARC* with the 42G allele of rs12075 was cloned between KpnI and XbaI sites of the vector to generate a ‘pcDNA3.1-DARC-42G’ construct. The fragment of *CCBP2* with the 373S allele of rs2228468 was cloned between KpnI and EcoRI sites of the vector to generate a ‘pcDNA3.1-D6-373S’ construct. A site-directed mutagenesis kit (Stratagene, USA) was used to generate the ‘pcDNA3.1-DARC-42D’ and ‘pcDNA3.1-D6-373Y’ constructs, respectively. Both constructs were confirmed by sequencing.

### Generation of stable transfectants

MDA-MB-231 cells were transfected with the same dose of plasmids or plasmid mixtures (1∶1) for transient transfection, respectively. Stable transfectants were selected by G418 (Invitrogen, USA) and identified by RT-PCR, real-time PCR, and western blot. The procedure of generation of stable transfectants using plasmid mixture and selection by G418 has been described elsewhere [22]. We screened and selected the transfectants expressing similarly high levels of DARC and/or D6 for further experiments.

Cell proliferation was done by using Cell Counting Kit-8 (Dojindo). Invasion experiments were conducted with a Matrigel invasion chamber (BD Labware). Flow cytometry analysis of DNA content was done to assess the cell cycle phase distribution. Due to limited number of words, the descriptions of DNA/RNA preparation, transient transfection, RT-PCR, real-time PCR, western blot, immunohistochemistry, and enzyme-linked immunosorbent assay (ELISA), are supplied in Text S1.

### Animal experiments

Four- to six-week-old athymic female BALB/c *nu*/*nu* mice were used in this study. A cohort of seventy nude mice was divided into seven groups of ten mice each. Cells (2×10^6^) were inoculated into the anesthetized mice in 100 µl of culture medium. The tumorigenicity of the cell lines was determined by injection into the cleared mammary fat pad of the mice. The volume of tumor was calculated by the following formula: volume = 0.5×length×width^2^. The mice were examined weekly for tumor appearance and growth. For ethical reasons, the primary tumors were surgically removed while mice were under Ketaset-Rompun anesthesia at five-weeks postinoculation. Mice were then maintained to allow further growth of lung metastases. Mice were sacrificed and autopsied at 12 weeks after initial tumor cell inoculation. DARC and D6 expression levels in the xenografts were assessed by western blot. The lung tissues were cut into 5 µm slices, and one in every ten sections was stained with H&E to evaluate the formation of lung metastasis.

### Isolation of human erythrocytes and erythrocytes chemokine sequestration assay

Within two hours of the whole blood collection, erythrocytes were purified using a technique as previously described [23]. Briefly, whole blood from patients with early-stage primary breast cancer was obtained via venipuncture and collected in K_2_-EDTA tubes (BD, Biosciences, USA). About 1 ml of the fresh whole blood was passed over a 5 ml syringe packed with a 2 ml column containing a mixture of microcrystalline cellulose (type 50) and α-cellulose (1∶1 by weight; Sigma-Aldrich, USA). The column was washed with 9 ml of sterile phosphate-buffered saline (PBS). The eluent was centrifuged at 1000 g for 4 minutes at 4°. The erythrocyte pellet was washed 3 times with cool, sterile PBS. The erythrocyte concentrates were stored at 4° in 50 ml polypropylene tubes (BD, Biosciences, USA). MDA-MB-231 cells were routinely cultured for 24 hours before the erythrocytes chemokine sequestration experiments. Freshly isolated erythrocytes from breast cancer patients with known genotypes were immediately counted manually. Conditioned media from the MDA-MB-231 cells were then incubated with 2×10^7^ erythrocytes or PBS at 37° for 1 hour. Erythrocytes were pelleted by centrifugation. The supernatant was collected and frozen at −80°.

### Statistical analysis

A Student's test or Mann-Whitney test was used to compare continuous variables between the two groups. Tests of association were conducted using the Pearson's χ2 test. The odds ratio (OR) and its 95% confidence intervals (CIs) were also determined. The significance levels of single locus association results were corrected using a false discovery rate (FDR) [24]. In FDR, as soon as one voxel (in this ascending list of p-value sorted voxels) is found that does not meet with the correction criterion, then all subsequent voxels are assumed to belong to the falsely claimed active voxels. Multivariate logistic regression was used to study the association between a single locus and the risk of LNM by adjusting for clinicopathological factors (method: forward stepwise, likelihood ratio). A *P*-value of <0.05 (two-sided) was considered statistically significant. Statistical analysis was performed using Stata/SE version 10.0 (StataCorp, USA) and SPSS Software version 12.0 (SPSS, USA).

## Results

### Subject characteristics

The clinicopathological characteristics of the breast cancer patients included in this study are summarized in **Table 1**, which shows that the mean age in the group with LNM was about two years lower than that in the group without LNM (*P*<0.001). Patients with LNM had a larger tumor size (*P*<0.001), a higher frequency of poorly differentiated tumor (*P*<0.001), and a higher proportion of hormone receptor negative tumor (*P*<0.001) and HER2 positive tumor (*P*<0.001) compared to those without LNM.

**Table 1:** Clinicopathological characteristics of the breast cancer patients.

Characteristics		Number (%)		*P*
		LN− (n = 785)	LN+ (n = 678)	
Age	years; mean±SD	50.8±10.5	48.6±9.7	<0.001
Tumor size	≤2 cm	484 (61.7)	268 (39.5)	<0.001*
	2–5 cm	262 (33.4)	316 (46.6)	
	>5 cm	30 (3.8)	75 (11.1)	
	Unknown	9 (1.1)	19 (2.8)	
ER	Positive	638 (81.3)	471 (69.5)	<0.001*
	Negative	138 (17.6)	188 (27.7)	
	Unknown	9 (1.1)	19 (2.8)	
PR	Positive	592 (75.5)	439 (64.8)	<0.001*
	Negative	184 (23.4)	220 (32.4)	
	Unknown	9 (1.1)	19 (2.8)	
HER2	Positive	166 (21.2)	272 (40.1)	<0.001*
	Negative	610 (77.7)	387 (57.1)	
	Unknown	9 (1.1)	19 (2.8)	
Grade	I	125 (15.9)	42 (6.2)	<0.001*
	II	493 (62.9)	376 (55.5)	
	III	158 (20.1)	241 (35.5)	
	Unknown	9 (1.1)	19 (2.8)	
Pathology	IDC	699 (89.1)	601 (88.7)	0.408
	ILC	65 (8.3)	51 (7.5)	
	Others	21 (2.6)	26 (3.8)	

SD, standard deviation; LN+, positive lymph node; LN−, negative lymph node; IDC, invasive ductal carcinoma; ILC, invasive lobular carcinoma; ER, estrogen receptor; PR, progesterone receptor; HER2, human epidermal growth factor receptor-2.

* based on a two-sided *χ* ^2^ test excluding the missing values.

### Selection of genetic variants and genotyping

Because of the limited data available on Chinese population *DARC* genetic variants in the HapMap database, we screened polymorphisms across the *DARC* genetic region and its flanking sequences (from approximately 1.0-kb upstream to 0.5-kb downstream of *DARC*) by directly sequencing the PCR products of genomic DNAs from the blood samples of 24 patients with sporadic breast cancer. This sub-sample was randomly selected from the total sample pool of our study. The primers used for identifying *DARC* SNPs are listed in **Table S2**. As a result, two polymorphisms, rs3027012, with a minor allele frequency (MAF) of 0.021 in 5′-flanking region, and rs12075 (G42D) with a MAF of 0.062 in exon 2, were identified (**Figure S1A**). SNP rs2814778, which was identified in the other ethnic samples previously, was not found in our sub-sample of 24 subjects (21, 22). Considering the potent biological function of rs2814778 (also recorded as T-46C, a mutation in *DARC* promoter disrupting a GATA consensus binding site), it was also selected for further genotyping within a much larger sample.

For *CCBP2*, SNPs were surveyed in the region spanning 59.3-kb from 1.0-kb upstream to 0.5-kb downstream of the transcribed sequence of *CCBP2* in the NCBI-dbSNP and the International HapMap websites. The HapMap database of the Han Chinese population (HapMap Data Rel 27/phase II+III) was used. Tagging-SNPs (tSNPs) were selected using the pairwise method under the restriction of MAFs>0.05 and *r* ^2^≥0.8, with the aim of identifying a set of tSNPs that efficiently captures all known common variants and that is likely to tag most unknown variants. In all, ten tSNPs were identified (rs9815043, rs4682859, rs3732859, rs4682857, rs13093968, rs1427804, rs17074834, rs17317763, rs7653015, and rs4683335) that capture all of the 33 common SNPs with a mean *r* ^2^ of 0.972. Among them, six tSNPs only tag themselves (rs13093968, rs1427804, rs17074834, rs17317763, rs7653015, and rs4683335). Each of these six SNPs was located in an intron and was thus excluded from further genotyping. Finally, we chose four representative tSNPs effectively capturing 27/33 (82%) common SNPs. Another SNP rs4683342 that was tagged by rs9815043 with a high *r* ^2^ was also included in further genotyping to technologically verify the genotyping results if rs9815043 failed in genotyping. Additionally, variants in *CCBP2* that may have functional effects (such as causing amino acid changes or alternative splicing) were chosen for genotyping whenever possible. Two potentially functional polymorphisms with MAFs>0.01 were used; they consisted of the SNP rs2228468 (Ser373Tyr, S373Y) and rs1366046 (in the 3′-untranslated region [UTR]) (**Figure S1B**). The plot of pairwise linkage disequilibrium (LD) among selected variants in the *CCBP2* gene is shown in **Figure S1C**.

In total, ten SNPs (three in *DARC* and seven in *CCBP2*) were selected for further genotyping. Genotyping work was performed using the 12-plex SNPstream system and was done by the Chinese National Human Genome Center in Shanghai. The sequences of the primers and probes for each SNP are listed in **Table S3**. In addition, 10% of the samples were randomly selected for re-genotyping for each of the ten SNPs and the results were 100% concordant.

### Association between LNM and SNPs in CDRs

Among the 10 SNPs selected for genotyping, no variation was observed for rs2814778 and therefore this SNP was excluded from the analysis. The remaining nine SNPs were successfully genotyped with genotyping call rates ranging from 95.0% to 99.7%.

There were significant associations between LNM and four of the studied SNPs, i.e., rs3027012 and rs12075 in *DARC*, and rs2228468 and rs1366046 in *CCBP2*, with the minor alleles having lower frequencies in the LNM patients compared with those without LNM (**Table 2**). After correction of multiple comparisons by FDR, the non-synonymous SNP, rs12075 (G42D), maintained a significant association with LNM (*P* = 4.0×10^−4^), and another non-synonymous SNP, rs2228468 (S373Y), remained borderline statistically significantly associated (*P* = 0.052). Compared with the G-allele, the A-allele of rs12075 could decrease the possibility of LNM by 50% (OR = 0.50, 95% CI, 0.35–0.69). Similar to results in the allelic genotype analysis, the data from genotype analysis indicated that the genotypes of rs3027012 and rs12075 in *DARC* and of rs2228468 in *CCBP2* were significantly correlated with LNM in a dominant model (*P* = 0.035, *P* = 3.36×10^−5^, and *P* = 0.027, respectively, **Table 2**), but not in a recessive model (data not shown).

**Table 2:** Allele and genotype frequencies of polymorphisms in *DARC* and *CCBP2* in relation to lymph node metastasis.

Gene	SNP	Allele	Location	Number§ (%)		OR (95%CI)*	*P* *	*P* **	OR (95% CI)#	*P* #
				LN−	LN+					
*DARC*	rs3027012	C	5′ near region	1523 (98.4)	1338 (99.3)	Reference	0.036	0.108		
		T		25 (1.6)	10 (0.7)	0.46 (0.22–0.95)				
		CC		749 (96.8)	664 (98.5)	Reference			Reference	
		TC		25 (3.2)	10 (1.5)	0.45 (0.22–0.95)	0.035		0.51 (0.23–1.14)	0.099
		TT		0 (0)	0 (0)	N.A.	N.A.		N.A.	
		TC+TT		250 (3.2)	10 (1.5)	0.45 (0.22–0.95)	0.035		0.51 (0.23–1.14)	0.099
*DARC*	rs12075	G	exon 2 (G42D)	1412 (92.4)	1253 (96.1)	Reference	4.7×10^−5^	4.0×10^−4^		
		A		116 (7.6)	51 (3.9)	0.50 (0.35–0.69)				
		GG		649 (84.9)	601 (92.2)	Reference			Reference	
		GA		114 (14.9)	51 (7.8)	0.48 (0.34–0.68)	4.25×10^−5^		0.60 (0.41–0.87)	0.007
		AA		1 (0.1)	0 (0.0)	N.A.	N.A		N.A.	
		GA+AA		115 (15.1)	51 (7.8)	0.48 (0.34–0.68)	3.36×10^−5^		0.59 (0.41–0.86)	0.006
*CCBP2*	rs4682857	C	intron 1	1300 (83.8)	1098 (84.1)	Reference	0.82	N.S.		
		G		252 (16.2)	208 (15.9)	0.98 (0.80–1.19)				
		CC		544 (70.1)	464 (71.1)	Reference			Reference	
		CG		212 (27.3)	170 (26.0)	0.94 (0.74–1.19)	0.61		0.83 (0.64–1.08)	0.164
		GG		20 (2.6)	19 (2.9)	1.11 (0.59–2.11)	0.74		1.06 (0.53–2.14)	0.870
		CG+GG		232 (29.9)	189 (28.9)	0.96 (0.76–1.20)	0.69		0.86 (0.67–1.10)	0.223
*CCBP2*	rs4682859	G	intron 2	948 (63.6)	837 (64.9)	Reference	0.49	N.S.		
		A		542 (36.4)	453 (35.1)	0.95 (0.81–1.11)				
		GG		295 (39.6)	262 (40.6)	Reference			Reference	
		GA		358 (48.1)	313 (48.5)	0.98 (0.79–1.23)	0.89		0.92 (0.72–1.18)	0.519
		AA		92 (12.3)	70 (10.9)	0.86 (0.60–1.22)	0.39		0.83 (0.57–1.21)	0.331
		GA+AA		450 (60.4)	383 (59.4)	0.96 (0.77–1.19)	0.70		0.90 (0.71–1.14)	0.393
*CCBP2*	rs4683342	C	intron 2	969 (63.1)	852 (64.9)	Reference	0.30	N.S.		
		G		567 (36.9)	460 (35.1)	0.92 (0.79–1.08)				
		CC		299 (38.9)	269 (41.0)	Reference			Reference	
		CG		371 (48.3)	314 (47.9)	0.94 (0.75–1.18)	0.59		0.88 (0.69–1.13)	0.313
		GG		98 (12.8)	73 (11.1)	0.83 (0.59–1.17)	0.28		0.66 (0.46–0.97)	0.033
		CG+GG		469 (61.1)	387 (59.0)	0.92 (0.74–1.13)	0.43		0.83 (0.66–1.05)	0.125
*CCBP2*	rs9815043	G	intron 2	1073 (69.1)	950 (71.1)	Reference	0.25	N.S.		
		A		479 (30.9)	386 (28.9)	0.91 (0.78–1.07)				
		GG		361 (46.5)	325 (48.7)	Reference			Reference	
		GA		351 (45.2)	300 (44.9)	0.95 (0.77–1.18)	0.64		0.91 (0.72–1.15)	0.425
		AA		64 (8.2)	43 (6.4)	0.75 (0.49–1.13)	0.17		0.62 (0.40–0.98)	0.040
		GA+AA		415 (53.5)	343 (51.3)	0.92 (0.75–1.13)	0.42		0.86 (0.69–1.08)	0.197
*CCBP2*	rs3732859	A	exon 3	1477 (94.3)	1284 (95.3)	Reference	0.26	N.S.		
		G		89 (5.7)	64 (4.7)	0.83 (0.59–1.15)				
		AA		696 (88.9)	611 (90.7)	Reference			Reference	
		GA		85 (10.9)	62 (9.2)	0.83 (0.59–1.17)	0.29		0.61 (0.43–8.52)	0.711
		GG		2 (0.3)	1 (0.1)	0.57 (0.05–6.30)	0.65		0.75 (0.52–1.09)	0.132
		GA+AA		87 (11.1)	63 (9.3)	0.82 (0.59–1.16)	0.27		0.75 (0.52–1.08)	0.124
*CCBP2*	rs2228468	G	exon 3 (S373Y)	1076 (69.0)	993 (73.2)	Reference	0.0116	0.052		
		T		484 (31.0)	363 (26.8)	0.81 (0.69–0.95)				
		GG		363 (46.5)	355 (52.4)	Reference			Reference	
		TG		350 (44.9)	283 (41.7)	0.83 (0.67–1.02)	0.082		0.80 (0.63–1.01)	0.061
		TT		67 (8.6)	40 (5.9)	0.61 (0.40–0.93)	0.021		0.53 (0.34–0.84)	0.007
		TG+TT		417 (53.5)	323 (47.6)	0.79 (0.64–0.97)	0.027		0.76 (0.60–0.95)	0.014
*CCBP2*	rs1366046	G	3′UTR	1075 (68.8)	979 (72.2)	Reference	0.0464	0.104		
		T		487 (31.2)	377 (27.8)	0.85 (0.72–1.00)				
		GG		358 (45.8)	345 (50.9)	Reference			Reference	
		TG		359 (46.0)	289 (42.6)	0.84 (0.67–1.03)	0.10		0.81 (0.64–1.02)	0.070
		TT		64 (8.2)	44 (6.5)	0.71 (0.47–1.08)	0.11		0.59 (0.38–0.93)	0.022
		TG+TT		423 (54.2)	333 (49.1)	0.82 (0.66–1.00)	0.05		0.77 (0.62–0.97)	0.023

LN+, positive lymph node; LN−, negative lymph node; OR, odds ratio; CI, confidence interval; UTR, untranslated region; N.S., not significant; N.A., not applicable.

§ some cases failed to be genotyped and these genotype data were thus missing.

* unadjusted *P*-value of two-sided *χ* ^2^ tests.

** corrected for multiple testing by false discovery rate.

# calculated by logistic regression, adjusted for age, tumor size, ER, PR, HER2, grade, and histology type.

Finally, we evaluated the predictive value of the four crude significant SNPs in *DARC* and *CCBP2* for the risk of LNM. Traditional variables of LNM also were chosen for the regression model. We found that rs12075 in *DARC* and rs2228468 in *CCBP2* were independent risk factors for LNM, with ORs (95% CI) of 0.54 (0.37–0.79) and 0.78 (0.62–0.98), respectively (**Table 3**).

**Table 3:** Multivariate logistic regression with the four SNPs in *DARC* and *CCBP2* and clinical risk factors for lymph node metastasis.

Risk factors for lymph node metastasis	OR (95% CI)	*P*
Age (continuous)	0.98 (0.97–0.99)	8.6×10^−4^
Size (≤2 cm vs. 2–5 cm vs. >5 cm)	2.05 (1.70–2.48)	8.0×10^−14^
Grade (I vs. II vs. III)	1.88 (1.54–2.30)	6.8×10^−10^
ER (Neg. vs. Pos.)	0.72 (0.55–0.96)	0.024
HER2 (Neg. vs. Pos.)	2.33 (1.81–2.99)	6.1×10^−11^
Pathology (others vs. ILC vs. IDC)		N.S.
PR (Neg. vs. Pos.)		N.S.
*DARC*-rs12075 (wt vs. vt&)	0.54 (0.37–0.79)	0.001
*CCBP2*-rs2228468 (wt vs. vt&)	0.78 (0.62–0.98)	0.031
*DARC*-rs3027012 (wt vs. vt&)		N.S.
*CCBP2*-rs1366046 (wt vs. vt&)		N.S.

IDC, invasive ductal carcinoma; ILC, invasive lobular carcinoma; wt, wild type; vt, variant type; N.S., not significant; Pos., positive; Neg., negative.

& comparing wild type with variant type in the dominant model.

### Significant SNPs in *DARC* and *CCBP2* do not have differential biological effects on gene expression *in vitro*

Although the two SNPs in *DARC* and *CCBP2* were associated with LNM in our epidemiological study, the precise mechanism is still unclear. We therefore examined whether the *DARC*-42G and *DARC*-42D alleles or the *CCBP2*-373S and *CCBP2*-373Y alleles had differential effects on DARC and D6 expression, respectively. No difference in expression was found between *DARC*-42G and *DARC*-42D or between *CCBP2*-373S and *CCBP2*-373Y in breast cancer cells, respectively (RT-PCR in **Figure S2A**, real-time PCR in Figure S2B, and western blot in **S2C**). No interaction effect of these two SNPs on gene expression was observed either.

We also generated stable transfectants of DARC. In this procedure, we aimed to obtain stable transfectants that expressed similarly high DARC despite having different alleles (42G and 42D). Similarly, we generated stable transfectants of D6 with different alleles (373S and 373Y). Because we had demonstrated that both of these SNPs (G42D and S373Y) had no effect on the differential expression of genes, the similar expression between *DARC*-42D and *DARC*-42G (or *CCBP2*-373Y and *CCBP2*-373S) in stable transfectants indicated that there was a comparable number of vector copies integrated into the genome of the MDA-MB-231 cells. Individual clones expressing similarly high levels of DARC and/or D6 were selected for further experiments.

In addition, to investigate whether these two SNPs could modulate the proliferation and invasion of MDA-MB-231 cells *in vitro*, we assessed the growth and invasion of *DARC*-42D and *DARC*-42G (or *CCBP2*-373Y and *CCBP2*-373S) transfectants. However, neither *DARC*-42G nor *CCBP2*-373S had an effect on the proliferation and invasion of cells compared to their wild-type counterpart. Moreover, no obvious changes in the cell cycle distribution could be seen between wild-type allele and variant allele for either *DARC* or *CCBP2*,

### Significant SNPs in *DARC* and *CCBP2* alter the chemokine sequestration ability of their corresponding proteins *in vitro*

Previous research in our lab has demonstrated that high-expression of DARC or D6 in human breast cancer cells induces inhibition of tumorigenesis and metastasis by clearing pro-malignant chemokines. We therefore examined whether the variants G42D and S373Y had any differential effects on chemokine sequestration ability. Similar to our previous results, the CXCL1, CXCL8, CCL2, and CCL5 levels were significantly lower in both the 231-DARC-42G and 231-DARC-42D transfectants compared with the controls (*P*<0.05). Similarly, the CCL2, CCL17, and CCL22 levels were significantly lower in both the 231-D6-373S and 231-D6-373Y transfectants compared with the control cells (*P*<0.05). Regarding the effect of G42D, the 231-DARC-42D transfectant had an additional 30–35% decrease in the levels of CXCL8, CCL2, and CCL5 relative to the 231-DARC-42G transfectant (*P* = 0.031, *P* = 0.028, and *P* = 0.027, respectively). Regarding S373Y, the 231-D6-373Y transfectant had an additional 16.4% decrease in the CCL2 level relative to the 231-D6-373S transfectant, with a borderline *P*-value of 0.069 (**Table 4**).

**Table 4:** Chemokine levels in the supernatant of the cells detected by ELISA after 24-hour incubation.

Concentration (pg/ml)	MDA-MB-231	231-vector	231-DARC-42G	231-DARC-42D	*P* *	231-D6-373S	231-D6-373Y	*P* #	231-DARC-42D-D6-373Y	*P* §
CXCL1	2447.0±95.3	2413.0±89.9	1795.0±80.0	1755.0±88.1	0.753	N.D.	N.D.	-	N.D.	-
CXCL8	568.3±18.8	560.0±17.3	271.0±21.9	177.7±18.2	0.031	N.D.	N.D.	-	N.D.	-
CCL2	280.3±11.6	274.3±11.6	183.3±13.0	127.7±10.1	0.028	219.0±9.5	183.3±10.9	0.069	54.9±6.3	<0.001
CCL5	25.0±1.4	25.7±1.3	18.2±1.2	12.6±1.1	0.028	25.4±1.4	24.5±1.2	0.665	8.41±1.0	<0.001
CCL17	238.3±7.3	238.7±7.9	N.D.	N.D.	-	135.0±8.7	111.7±9.3	0.140	N.D.	-
CCL22	425.6±13.2	428.7±13.9	N.D.	N.D.	-	326.7±11.7	303.3±13.0	0.253	N.D.	-

* comparison between two alleles of SNP G42D in *DARC*.

# comparison between two alleles of SNP S373Y in *CCBP2* (coding D6).

§ 231-DARC-42D-D6-373Y group compared with 231-vector group.

Columns, mean of three independent experiments with standard error.

N.D., not detected.

Having observed differential chemokine sequestration ability of each SNP, we further investigated the joint effect of rs12075 and rs2228468. These two SNPs had a synergistic effect on altering the chemokine sequestration capabilities of their CDR proteins. For instance, 231-DARC-42D and 231-D6-373Y decreased the CCL2 levels by 53% and 33%, respectively. When the 2 SNPs work together, the theoretical clearance rate for synthetic effect should be 69% (calculated by the formula: [53%+(1–53%)*33%]). Actually, the combination of the two proteins harboring the two SNPs (231-DARC-42D-D6-373Y) reduced the CCL2 level by 80%, much higher than 69%. A similar trend was observed in the CCL5 levels (**Table 4**).

### Significant SNPs in *DARC* and *CCBP2* have differential effects on inhibition of tumor growth, angiogenesis, and lung metastasis by interfering with chemokine sequestration ability *in vivo*

**Figure 1:**
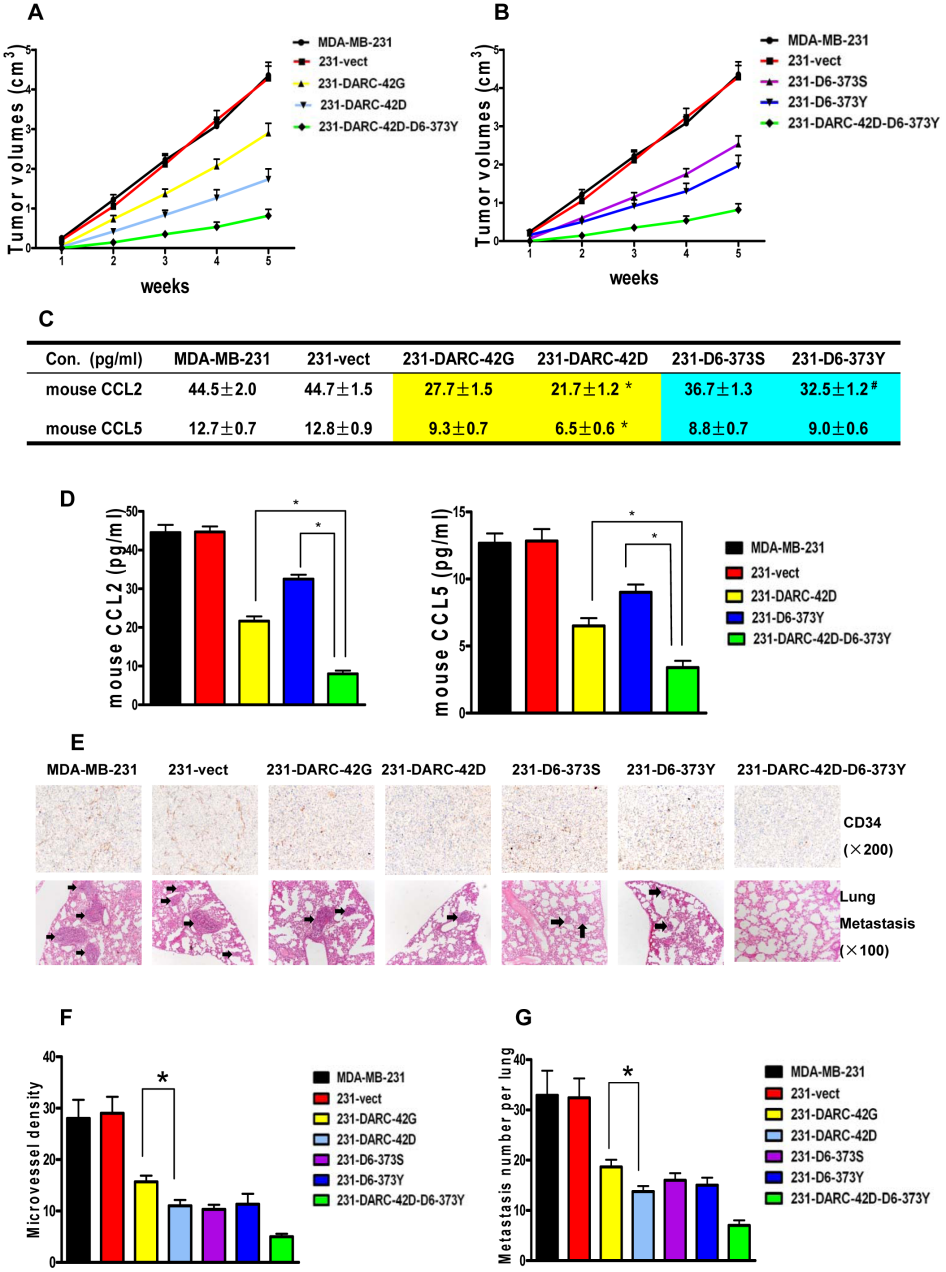
Differential effects on inhibition of tumor growth, angiogenesis and lung metastasis by the significant SNPs that interfere with chemokine sequestration ability *in vivo*. **A–B**, The tumor growth curves. **C–D**, Chemokine levels in the xenografts of mice detected by ELISA: the grids in yellow indicate *P*<0.05 (comparison between the DARC highly expressive xenografts and the controls); the grids in blue indicate *P*<0.05 (comparison between the D6 highly expressive xenografts and the controls); * denotes *P*<0.05 and ^#^ denotes 0.05<*P*<0.10. Columns, mean of three independent experiments; error bars, standard error. **E**, Representative images of CD34 staining (×200, as a marker of microvessels) and the lung metastasis nodules (H&E, ×100, arrows). **F**, Quantification of the mean number of microvessels of the xenografts. *, *P*<0.05. **G**, quantification of the mean number of metastases of per lung. *, *P*<0.05.

The effects of the two significant SNPs on tumorigenicity and metastasis potential *in vivo* were further investigated by using an orthotropic xenograft tumor model in nude mice. Significantly higher DARC and D6 expression levels were observed in tumors formed by DARC and/or D6 high-expressing transfectants compared with the controls (**Figure S3**). The xenografts with either DARC or D6 high expression levels had slower growth than the controls (**Figure 1A and 1B**). The mean tumor volume was 2.90 cm^3^ for the 231-DARC-42G xenografts and was 1.73 cm^3^ for the 231-DARC-42D xenografts after five-weeks, suggesting that DARC-42D had a 27.4% higher effect on the inhibition of tumor proliferation relative to DARC-42G (*P* = 0.031). But the difference in the tumor proliferation-inhibiting effect between D6-373S and D6-373Y was not obvious. Furthermore, the mean tumor volume of the 231-DARC-42D-D6-373Y xenografts (0.82 cm^3^) is 19.1% as small as that of the 231-vect xenografts, suggesting that co-expression of DARC and D6 has a synergistic effect on the inhibition of tumor growth.

Moreover, the extent to which the local concentration of target chemokines within the xenograft tumors was influenced by the significant SNPs *in vivo* was analyzed. The mouse CCL2 and CCL5 levels within the DARC and D6 xenografts were significantly lower than those in the controls (*P*<0.05). The mouse CCL2 and CCL5 levels in the 231-DARC-42D xenografts were about 23–30% lower than those in the 231-DARC-42G xenografts (*P* = 0.034 and *P* = 0.038, respectively). Trending towards significance, the mouse CCL2 level in the 231-D6-373Y xenografts was about 11.4% lower than that in the 231-D6-373S xenografts (*P* = 0.075, **Figure 1C**). The synergistic effects of these two CDRs on the chemokine sequestration ability in xenograft tumors were also observed (**Figure 1D**).

The potential mechanisms involved in the retardation of tumor growth *in vivo* were examined. There were significantly fewer microvessels in the DARC and/or D6 high-expressing tumors than in the control tumors. In particular, the number of microvessels in the tumors formed by 231-DARC-42D was nearly 70% of the number formed by 231-DARC-42G. However, there was no difference in the microvessel counts between the tumors formed by 231-D6-373S and 231-D6-373Y. Tumors harboring both DARC and D6 had a synergistic effect on the inhibition of angiogenesis (**Figure 1E and 1F**).

The effects of the significant SNPs on ability to metastasize *in vivo* were also investigated. The incidences of lung metastasis were 10/10, 10/10, 6/10, 4/10, 5/10, 5/10, 2/10 for mice injected with MDA-MB-231, 231-vect, 231-DARC-42G, 231-DARC-42D, 231-D6-373S, 231-D6-373Y, and 231-DARC-42D-D6-373Y, respectively. Furthermore, mice injected with DARC and/or D6 high-expressing transfectants had significant fewer lung metastasis nodules than those with control cells. Similarly, the number of lung metastasis nodules in the mice injected with 231-DARC-42D was approximately 73% of the number of nodules in the mice injected with 231-DARC-42G (*P* = 0.037). There was not a significant difference in the numbers of lung metastasis nodules between the mice injected with 231-D6-373Y and those injected with 231-D6-373S. But a synergistic role in the inhibition of lung metastasis was also observed (**Figure 1E and 1G**).

### Variation G42D in DARC influences the chemokine sequestration ability of erythrocytes *ex vivo*

**Figure 2:**
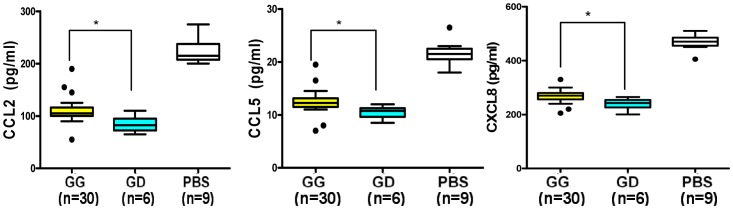
The G42D variant has an effect on chemokine sequestration ability of the DARC protein expressed on human erythrocytes. G42D altered the chemokine sequestration ability of erythrocytes *ex vivo*. Chemokine levels in the supernatant of breast cancer cells were detected by ELISA after incubation with erythrocytes that were harboring different genotypes or with PBS. * *P*<0.05 by the Mann-Whitney test. The horizontal lines represent the mean values.

DARC protein is an abundant receptor expressed on erythrocytes. The effect of rs12075 (G42D) on the chemokine sequestration ability of different genotype erythrocytes was examined *ex vivo*. The CCL2, CCL5, and CXCL8 levels were significantly lower in the supernatant of the conditioned media that had been incubated with the erythrocytes of the 42GD heterozygote than those incubated with the erythrocytes of the 42GG homozygote (*P* = 0.007, *P* = 0.004, and *P* = 0.008, **Figure 2**).

## Discussion

The present study was based on the hypothesis that SNPs in the genes encoding CDRs that determine pro-malignant chemokine concentrations should also be associated with breast cancer metastasis. Lymph nodes are often the first sites where breast cancer cells spread [25]. It is well established that LNM is an independent indicator of aggressive behavior of primary breast cancer [26]. Here we chose ‘lymph node involvement’ as the surrogate for ‘phenotype of metastasis’, and 10 SNPs in *DARC* and *CCBP2* were analyzed in a cohort of Han Chinese breast cancer patients to test our hypothesis. After a multivariate analysis, with adjustment for classic LNM risk factors, significant associations between two polymorphisms, rs12075 in *DARC* and rs2228468 in *CCBP2*, and the risk of LNM were found. Functional assays revealed that the DARC encoded by the *DARC*-42D allele had increased chemokine-sequestration ability compared with that encoded by the *DARC*-42G allele. Moreover, DARC-42D had an enhanced inhibitory effect on tumor growth and metastasis in xenograft models. The synergistic effect of chemokine sequestration ability was observed when DARC and D6 were combined. The results in our current study consistently suggest that rs12075 in *DARC* and rs2228468 in *CCBP2* are two important genetic determinants for breast cancer metastasis potential, mainly by regulating the pro-malignant chemokine levels in the tumor microenvironment.

Initially, DARC was introduced as the antigen of the Duffy blood group system that consists of two variants, Fy^a^ and Fy^b^, which are identified by the Gly42Asp polymorphism (rs12075, G42D) [27], [28]. Recently, this non-synonymous SNP was identified as a major determinant of circulating CCL2 concentration in a genome-wide association study [29]. But no data have been presented describing an association between SNPs in *CCBP2* and cancer initiation or progression. The biological function of the two non-synonymous SNPs (rs12075 in *DARC* and rs2228468 in *CCBP2*) in the regulation of cancer metastasis is largely unknown, and we proposed that the mechanism by which the two SNPs respectively alter DARC and D6 function may be mediated by amino acid substitution. In the functional assays, DARC-42D led to a higher ability of sequestering the pro-malignant chemokines than DARC-42G in tumor cell lines *in vitro* and in xenograft tumors *in vivo*. Furthermore, human erythrocytes carrying the DARC-42GD genotype in an *ex vivo* model showed significantly stronger chemokine sequestration ability than their counterparts carrying the DARC-42GG genotype. Additionally, microvessel density in DARC-42D expressing tumors is also lower than that in DARC-42G expressing tumors. Metastasis is a multi-step and complex process. Our experimental data suggest that DARC isoforms in the tumor microenvironment could reduce the levels of pro-malignant chemokines (such as CCL2, CCL5, and CXCL8) in different degrees, have differential effects on tumor growth and vascularization, and contribute to differential potential of metastasis. Taken together, the change in DARC function due to the non-synonymous SNP rs12075 may interfere with the metastatic potential of breast cancer via altered chemokine sequestration ability. Another non-synonymous SNP in *CCBP2*, rs2228468, showed limited differential effects on chemokine sequestration ability and metastatic potential. The biological function of this SNP should be further elucidated. Notably, it was evident that the combination of DARC and D6 displayed a synergistic effect on clearing CCL2 and CCL5. One reasonable explanation is that DARC and D6 have overlapping functions in the sequestration of CCL2 and CCL5, binding to these chemokines with high affinity [30], [31]. Therefore, DARC and D6 can act together to clear more pro-malignant chemokines. Alternatively, since CCL2 is central to the inflammatory process and it could up-regulate the release of CCL5 from endogenous pre-made vesicles in breast cancer cells, it is reasonable that the mouse tumors expressing both DARC and D6 significantly reduced mouse CCL2, thus resulting in significantly lower mouse CCL5 level detected in those tumors. Therefore, it is expected that the patients carrying certain multiple genotypes of CDR genes might have a critically modified chemokine network in the tumor microenvironment, thus being far less susceptible to breast cancer metastasis.

Our study has several limitations. First, LNM could be due to later stage at diagnosis and does not absolutely represent aggressiveness or potential for metastasis; there could be metastasis without lymphatic invasion. In this study, we chose LNM as the surrogate of a metastasis phenotype; this choice of endpoint for metastasis should be further justified. Second, this study has a relatively small sample size. The promising association between SNPs and the metastatic potential of breast cancer needs to be replicated in other large-scale, independent population sets. Third, immune cell infiltration in xenograft tumors generated from stable transfectants expressing different isoforms of DARC or D6 remains to be assessed. Despite of these limitations, our data suggest that rs12075 and rs2228468 act as indicators of altered metastatic potential of breast cancer in Han Chinese patients.

In summary, our study shows an association between rs12075 in *DARC* and rs2228468 in *CCBP2* and susceptibility to breast cancer metastasis. The two SNPs, which cause amino acid substitutions, might lead to differential chemokine sequestration ability, which may be the underlying mechanism that confers LNM risk. Although further investigation on detailed mechanism is still needed, our findings probably support the hypothesis that genetic polymorphisms in the genes encoding CDRs could mediate metastatic risk.

## Supporting Information

Text S1 **Supplemental Materials and Methods.**
(DOC)

Figure S1 **Candidate SNPs for genotyping in** ***DARC*** **and** ***CCBP2*** **.** **A**, Three SNPs were chosen for genotyping in the 3.2-kb region of *DARC*, from 1.0-kb upstream of the 5′-flanking region to 0.5-kb downstream of the 3′-flanking region. One SNP, rs2814778 (arrow in blue), was excluded because no polymorphism was detected in our subjects. **B**, Seven SNPs were chosen for genotyping in the 59.3-kb region of *CCBP2*, from 1 kb upstream of the 5′-flanking region to 0.5-kb downstream of the 3′-flanking region. **C**, Pairwise linkage disequilibrium (LD) among selected variants in the *CCBP2* gene. The value within each diamond represents the pairwise correlation between polymorphisms (measured as D' (×100)) defined by the upper left and the upper right sides of the diamond. The red-to-white gradient reflects higher to lower LD values, and the diamond without a number corresponds to D' = 1.
(PPT)

Figure S2 **Significant SNPs in** ***DARC*** **and** ***CCBP2*** **have no differential effect on gene expression but do have an effect on chemokine sequestration ability of their coding proteins** ***in vitro*** **.** **A**, Representative pictures of gene expression in transient transfectants detected by RT-PCR. **B**, Gene expression in transient transfectants detected by real-time PCR. **C**, Representative pictures of protein expression in transient transfectants detected by western blot. All transient transfection experiments were normalized for β-galactosidase activity. All experiments were repeated three times, and *GAPDH* was chosen as an internal control. The relative protein level in the different cell lines was normalized to the signal intensity of GAPDH. Lane 1, MDA-MB-231; lane 2, 231-vect; lanes 3–4, 231-DARC-42G; lanes 5–6, 231-DARC-42D; lanes 7–8, 231-D6-373S; lanes 9–10, 231-D6-373Y; lanes 11–12, 231-DARC-42D-D6-373Y; lanes 13–14, 231-DARC-42G-D6-373S.
(PPT)

Figure S3 **DARC and D6 expression levels in xenograft tumors.** Representative pictures of DARC and D6 expression levels in xenografts assessed by western blot: lane 1, MDA-MB-231; lane 2, 231-vect; lane 3, 231-DARC-42G; lane 4, 231-DARC-42D; lane 5, 231-DARC-42D-D6-373Y; lane 6, 231-D6-373S; lane 7, 231-D6-373Y.
(PPT)

Table S1 **Primers for the plasmid constructs, RT-PCR and real-time PCR.**
(DOC)

Table S2 **Primers for discovering SNPs in** ***DARC*** **.**
(DOC)

Table S3 **Primers and probes for the SNP Stream platform.**
(DOC)

## References

[1] Jemal A, Siegel R, Xu J, Ward E (2010) Cancer statistics, 2010. CA Cancer J Clin 60: 277–300. 20610543 10.3322/caac.20073

[2] Beckmann MW, Niederacher D, Schnurch HG, Gusterson BA, Bender HG (1997) Multistep carcinogenesis of breast cancer and tumour heterogeneity. J Mol Med 75: 429–439. 9231883 10.1007/s001090050128

[3] Balakin KV, Ivanenkov YA, Tkachenko SE, Kiselyov AS, Ivachtchenko AV (2008) Regulators of chemokine receptor activity as promising anticancer therapeutics. Curr Cancer Drug Targets 8: 299–340. 18537553 10.2174/156800908784533490

[4] Zlotnik A (2006) Chemokines and cancer. Int J Cancer 119: 2026–2029. 16671092 10.1002/ijc.22024

[5] Soria G, Ben-Baruch A (2008) The inflammatory chemokines CCL2 and CCL5 in breast cancer. Cancer Lett 267: 271–285. 18439751 10.1016/j.canlet.2008.03.018

[6] Soria G, Yaal-Hahoshen N, Azenshtein E, Shina S, Leider-Trejo L, et al (2008) Concomitant expression of the chemokines RANTES and MCP-1 in human breast cancer: a basis for tumor-promoting interactions. Cytokine 44: 191–200. 18790652 10.1016/j.cyto.2008.08.002

[7] Azenshtein E, Meshel T, Shina S, Barak N, Keydar I, et al (2005) The angiogenic factors CXCL8 and VEGF in breast cancer: regulation by an array of pro-malignancy factors. Cancer Lett 217: 73–86. 15596298 10.1016/j.canlet.2004.05.024

[8] Azenshtein E, Luboshits G, Shina S, Neumark E, Shahbazian D, et al (2002) The CC chemokine RANTES in breast carcinoma progression: regulation of expression and potential mechanisms of promalignant activity. Cancer Res 62: 1093–1102. 11861388

[9] Ghilardi G, Biondi ML, La Torre A, Battaglioli L, Scorza R (2005) Breast cancer progression and host polymorphisms in the chemokine system: role of the macrophage chemoattractant protein-1 (MCP-1) -2518 G allele. Clin Chem 51: 452–455. 15681563 10.1373/clinchem.2004.041657

[10] Snoussi K, Mahfoudh W, Bouaouina N, Ahmed SB, Helal AN, et al (2006) Genetic variation in IL-8 associated with increased risk and poor prognosis of breast carcinoma. Hum Immunol 67: 13–21. 16698420 10.1016/j.humimm.2006.03.018

[11] Snoussi K, Mahfoudh W, Bouaouina N, Fekih M, Khairi H, et al (2010) Combined effects of IL-8 and CXCR2 gene polymorphisms on breast cancer susceptibility and aggressiveness. BMC Cancer 10: 283. 20540789 10.1186/1471-2407-10-283 PMC2895614

[12] Peiper SC, Wang ZX, Neote K, Martin AW, Showell HJ, et al (1995) The Duffy antigen/receptor for chemokines (DARC) is expressed in endothelial cells of Duffy negative individuals who lack the erythrocyte receptor. J Exp Med 181: 1311–1317. 7699323 10.1084/jem.181.4.1311 PMC2191961

[13] Nibbs RJ, Wylie SM, Yang J, Landau NR, Graham GJ (1997) Cloning and characterization of a novel promiscuous human beta-chemokine receptor D6. J Biol Chem 272: 32078–32083. 9405404 10.1074/jbc.272.51.32078

[14] Gosling J, Dairaghi DJ, Wang Y, Hanley M, Talbot D, et al (2000) Cutting edge: identification of a novel chemokine receptor that binds dendritic cell- and T cell-active chemokines including ELC, SLC, and TECK. J Immunol 164: 2851–2856. 10706668 10.4049/jimmunol.164.6.2851

[15] Wang J, Ou ZL, Hou YF, Luo JM, Shen ZZ, et al (2006) Enhanced expression of Duffy antigen receptor for chemokines by breast cancer cells attenuates growth and metastasis potential. Oncogene 25: 7201–7211. 16785997 10.1038/sj.onc.1209703

[16] Wu FY, Ou ZL, Feng LY, Luo JM, Wang LP, et al (2008) Chemokine decoy receptor d6 plays a negative role in human breast cancer. Mol Cancer Res 6: 1276–1288. 18708360 10.1158/1541-7786.MCR-07-2108

[17] Feng LY, Ou ZL, Wu FY, Shen ZZ, Shao ZM (2009) Involvement of a novel chemokine decoy receptor CCX-CKR in breast cancer growth, metastasis and patient survival. Clin Cancer Res 15: 2962–2970. 19383822 10.1158/1078-0432.CCR-08-2495

[18] Rot A (2005) Contribution of Duffy antigen to chemokine function. Cytokine Growth Factor Rev 16: 687–694. 16054417 10.1016/j.cytogfr.2005.05.011

[19] Locati M, Torre YM, Galliera E, Bonecchi R, Bodduluri H, et al (2005) Silent chemoattractant receptors: D6 as a decoy and scavenger receptor for inflammatory CC chemokines. Cytokine Growth Factor Rev 16: 679–686. 15996892 10.1016/j.cytogfr.2005.05.003

[20] Yu KD, Di GH, Wu J, Lu JS, Shen KW, et al (2007) Development and trends of surgical modalities for breast cancer in China: a review of 16-year data. Ann Surg Oncol 14: 2502–2509. 17564750 10.1245/s10434-007-9436-2

[21] Yin WJ, Lu JS, Di GH, Lin YP, Zhou LH, et al (2009) Clinicopathological features of the triple-negative tumors in Chinese breast cancer patients. Breast Cancer Res Treat 115: 325–333. 18563552 10.1007/s10549-008-0096-0

[22] Jahroudi N, Ardekani AM, Greenberger JS (1996) Ionizing irradiation increases transcription of the von Willebrand factor gene in endothelial cells. Blood 88: 3801–3814. 8916944

[23] Beutler E, West C, Blume KG (1976) The removal of leukocytes and platelets from whole blood. J Lab Clin Med 88: 328–333. 956688

[24] Benjamini Y, Hochberg Y (1995) Controlling the false discovery rate: a practical and powerful approach to multiple testing. J R Statist Soc B 57: 289–300.

[25] Singletary SE, Connolly JL (2006) Breast cancer staging: working with the sixth edition of the AJCC Cancer Staging Manual. CA Cancer J Clin 56: 37–47 quiz 50-1. 16449185 10.3322/canjclin.56.1.37

[26] Rack B, Janni W, Gerber B, Strobl B, Schindlbeck C, et al (2003) Patients with recurrent breast cancer: does the primary axillary lymph node status predict more aggressive tumor progression? Breast Cancer Res Treat 82: 83–92. 14692652 10.1023/B:BREA.0000003955.73738.9e

[27] Tournamille C, Le Van Kim C, Gane P, Cartron JP, Colin Y (1995) Molecular basis and PCR-DNA typing of the Fya/fyb blood group polymorphism. Hum Genet 95: 407–410. 7705836 10.1007/BF00208965

[28] Mallinson G, Soo KS, Schall TJ, Pisacka M, Anstee DJ (1995) Mutations in the erythrocyte chemokine receptor (Duffy) gene: the molecular basis of the Fya/Fyb antigens and identification of a deletion in the Duffy gene of an apparently healthy individual with the Fy(a-b-) phenotype. Br J Haematol 90: 823–829. 7669660 10.1111/j.1365-2141.1995.tb05202.x

[29] Schnabel RB, Baumert J, Barbalic M, Dupuis J, Ellinor PT, et al (2010) Duffy antigen receptor for chemokines (Darc) polymorphism regulates circulating concentrations of monocyte chemoattractant protein-1 and other inflammatory mediators. Blood 115: 5289–5299. 20040767 10.1182/blood-2009-05-221382 PMC2902130

[30] Neote K, Mak JY, Kolakowski LF Jr, Schall TJ (1994) Functional and biochemical analysis of the cloned Duffy antigen: identity with the red blood cell chemokine receptor. Blood 84: 44–52. 7517217

[31] Kashiwazaki M, Tanaka T, Kanda H, Ebisuno Y, Izawa D, et al (2003) A high endothelial venule-expressing promiscuous chemokine receptor DARC can bind inflammatory, but not lymphoid, chemokines and is dispensable for lymphocyte homing under physiological conditions. Int Immunol 15: 1219–1227. 13679391 10.1093/intimm/dxg121

