# Supplementary material for: Effect of Genetic Variants in Two Chemokine Decoy Receptor Genes, DARC and CCBP2, on Metastatic Potential of Breast Cancer
Source: PLoS One. 2013 Nov 15;8(11):e78901. doi: 10.1371/journal.pone.0078901 (PMC3829817; doi:10.1371/journal.pone.0078901)
Supplement: Text S1 — Supplemental Materials and Methods. (DOC) [file pone.0078901.s001.doc]

**Supplementary Text-Materials and Methods**

**DNA/RNA preparation**

Genomic DNA was extracted from 3 to 5 ml of the study participant’s peripheral blood leukocytes using the PureGene DNA Purification kit (Gentra Systems, USA) according to the manufacturer’s protocol, and then stored at -20°. Cancer-free breast tissue samples (n=45) were obtained surgically from women who were receiving biopsies; the samples that were pathologically confirmed in our department to have fibrocystic changes were snap frozen in liquid nitrogen and stored at -80°. mRNA was extracted from frozen breast tissue and from cultured cells using the TRIzol Reagent (Invitrogen, USA) before being reverse transcribed. General PCR was performed as described previously .

**Transient transfection**

In single plasmid transfection, 1 μg of the expression plasmid pcDNA3.1 with a *DARC* or *CCBP2* fragment were transfected using Lipofectamine 2000 (Invitrogen, USA), according to the manufacturer’s instruction. In co-transfection experiments, the combination of 1 μg pcDNA3.1-DARC-42D and 1 μg pcDNA3.1-D6-373Y, or the combination of 1 μg pcDNA3.1-DARC-42G and 1 μg pcDNA3.1-D6-373S were transfected together. An empty expression vector was also transfected into MDA-MB-231 cells to generate a control cell line (named 231-vect). 0.2 μg β-galactosidase expression plasmid pCMV-β-galactosidase was co-transfected to normalize for variations in transfection efficiency. All transient transfection experiments were carried out in triplicate, repeated at least three times, and normalized for β-galactosidase activity. Expression of DARC and/or D6 was analyzed by RT-PCR, real-time PCR, and western blot techniques.

**RT-PCR**

RT-PCR was performed according to the manufacturer’s instructions (MBI Fermentas, USA). The specific primers for *DARC* and *CCBP2* used to analyze stable transfectants are listed in **Supplementary Table S3**.

**Real-time PCR**

Extracted mRNA was subjected to real-time PCR with the SYBR Green fluorescent-based assay (TaKaRa, Japan), as previously described [2], in a fluorescence temperature cycler (Opticon, MJ Research, USA), using the modified 2^–△△Ct^ method [3]. The primers and PCR conditions are described in **Supplementary Table S3**.

**Western blot**

Western blots using goat anti-human polyclonal DARC antibody (Abcam, USA) or goat anti-human polyclonal D6 antibody (Abcam, USA) were performed according to the standard protocol [4]. Images were analyzed by Quantity One software (Bio-rad, USA).

**Immunohistochemistry detection of microvessels**

Tumor sections were subjected to immunohistochemical staining for CD34. Tumor sections were incubated in a 1:50 diluton of rat anti-mouse CD34 (Abcam, USA). Primary antibodies were detected with HRP-conjugated secondary antibodies followed by colorimetric detection with 3,3-diaminobenzidine (DAB). CD34-stained sections were scanned at low magnification (×100) to determine the areas with the highest number of microvessels (hot spots). Microvessels whose diameters were less than that of eight red cells were counted at a magnification of ×200 in two hot spots on each section, and microvessel density (MVD) was calculated as the average of the two measurements.

**Enzyme-linked immunosorbent assay (ELISA)**

The protein levels of human CXCL1, CXCL8, CCL2, CCL5, CCL17, and CCL22 that were present in the conditioned cell supernatants or in the supernatants collected from the erythrocyte chemokine sequestration assay, as well as the mouse CCL2 and CCL5 levels in the xenografts, were determined with a sandwich ELISA kit (R&D systems, USA).

**References**

1. Li WF, Hu Z, Rao NY, Song CG, Zhang B, et al. (2008) The prevalence of BRCA1 and BRCA2 germline mutations in high-risk breast cancer patients of Chinese Han nationality: two recurrent mutations were identified. Breast Cancer Res Treat 110: 99-109.

2. Aldea C, Alvarez CP, Folgueira L, Delgado R, Otero JR. (2002) Rapid detection of herpes simplex virus DNA in genital ulcers by real-time PCR using SYBR green I dye as the detection signal. J Clin Microbiol 40:1060-1062.

3. Pfaffl MW (2001) A new mathematical model for relative quantification in real-time RT-PCR. Nucleic Acids Res 29 :e45.

4. Li DQ, Wang L, Fei F, Hou YF, Luo JM, et al. (2006) Identification of breast cancer metastasis-associated proteins in an isogenic tumor metastasis model using two-dimensional gel electrophoresis and liquid chromatography-ion trap-mass spectrometry. Proteomics 6: 3352 -3368.
