# Supplementary figures and images for: Effect of Genetic Variants in Two Chemokine Decoy Receptor Genes, DARC and CCBP2, on Metastatic Potential of Breast Cancer
Source: PLoS One. 2013 Nov 15;8(11):e78901. doi: 10.1371/journal.pone.0078901 (PMC3829817; doi:10.1371/journal.pone.0078901)

## Slide 1
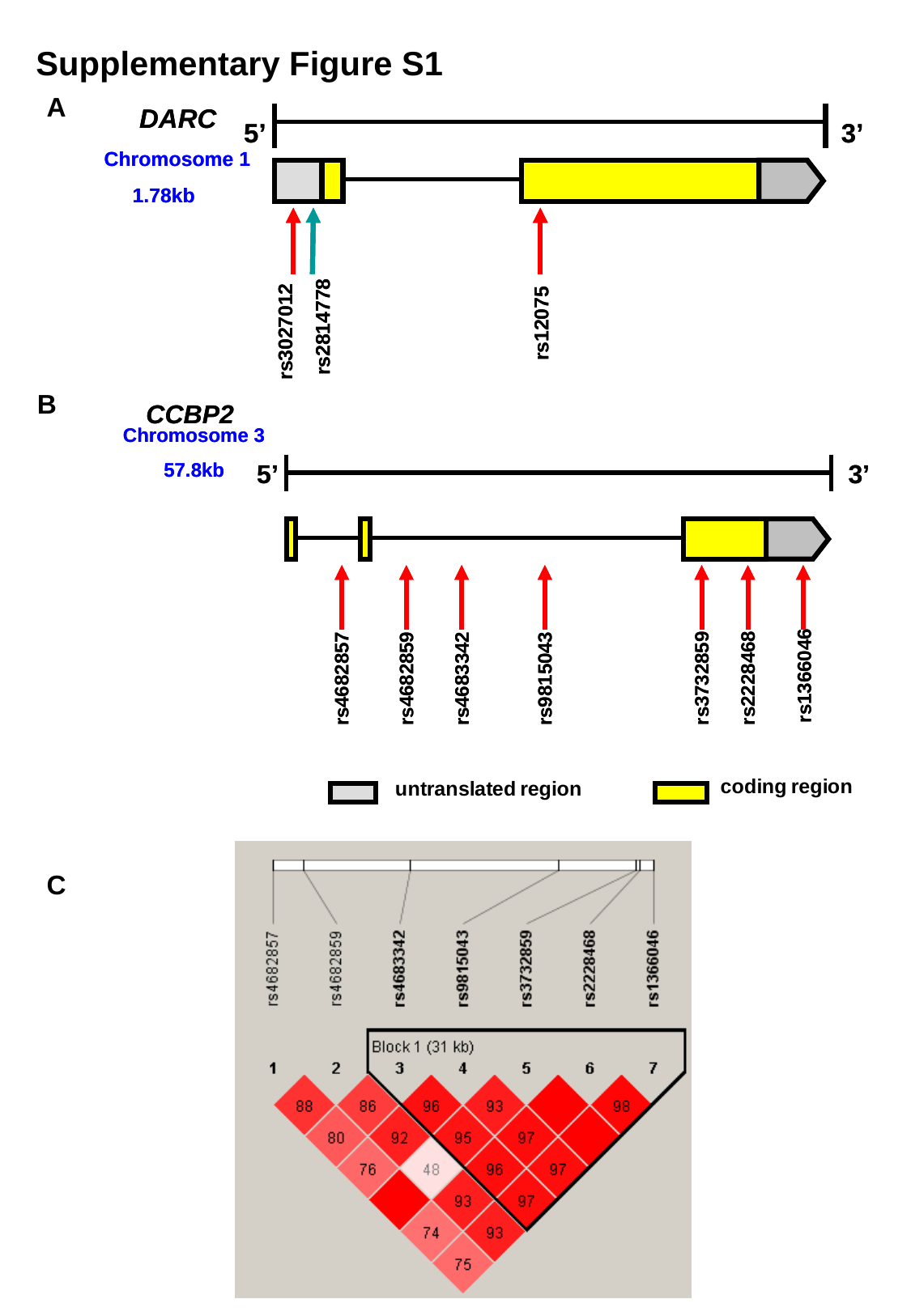

Supplementary Figure S1
A
B
C

Supplement: Figure S1 — Candidate SNPs for genotyping in DARC and CCBP2 . A, Three SNPs were chosen for genotyping in the 3.2-kb region of DARC, from 1.0-kb upstream of the 5′-flanking region to 0.5-kb downstream of the 3′-flanking region. One SNP, rs2814778 (arrow in blue), was excluded because no polymorphism was detected in our subjects. B, Seven SNPs were chosen for genotyping in the 59.3-kb region of CCBP2, from 1 kb upstream of the 5′-flanking region to 0.5-kb downstream of the 3′-flanking region. C, Pairwise linkage disequilibrium (LD) among selected variants in the CCBP2 gene. The value within each diamond represents the pairwise correlation between polymorphisms (measured as D' (×100)) defined by the upper left and the upper right sides of the diamond. The red-to-white gradient reflects higher to lower LD values, and the diamond without a number corresponds to D' = 1. (PPT) [file pone.0078901.s002.ppt]

## Slide 1
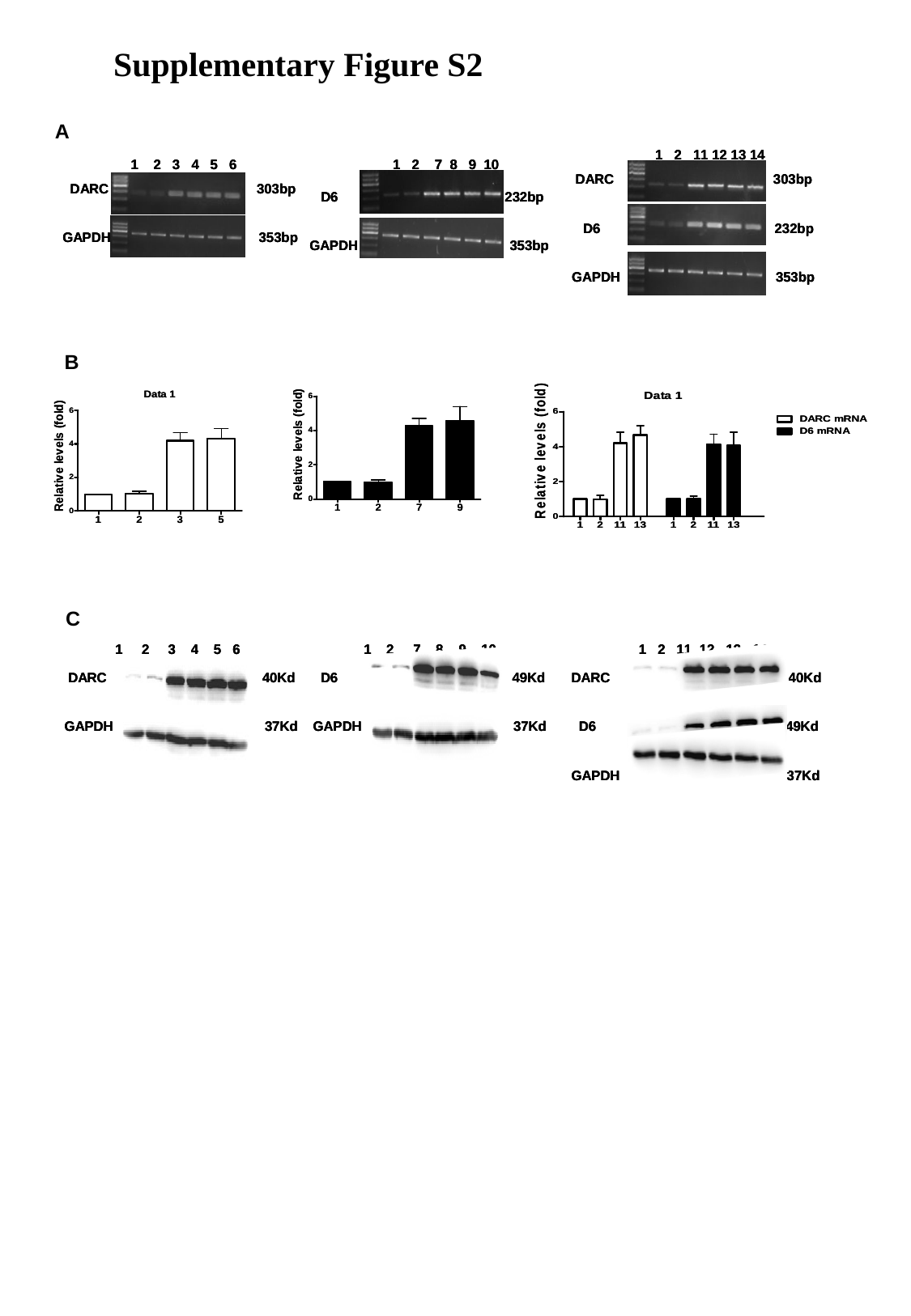

Supplementary Figure S2
A
B
C

Supplement: Figure S2 — Significant SNPs in DARC and CCBP2 have no differential effect on gene expression but do have an effect on chemokine sequestration ability of their coding proteins in vitro . A, Representative pictures of gene expression in transient transfectants detected by RT-PCR. B, Gene expression in transient transfectants detected by real-time PCR. C, Representative pictures of protein expression in transient transfectants detected by western blot. All transient transfection experiments were normalized for β-galactosidase activity. All experiments were repeated three times, and GAPDH was chosen as an internal control. The relative protein level in the different cell lines was normalized to the signal intensity of GAPDH. Lane 1, MDA-MB-231; lane 2, 231-vect; lanes 3–4, 231-DARC-42G; lanes 5–6, 231-DARC-42D; lanes 7–8, 231-D6-373S; lanes 9–10, 231-D6-373Y; lanes 11–12, 231-DARC-42D-D6-373Y; lanes 13–14, 231-DARC-42G-D6-373S. (PPT) [file pone.0078901.s003.ppt]

## Slide 1
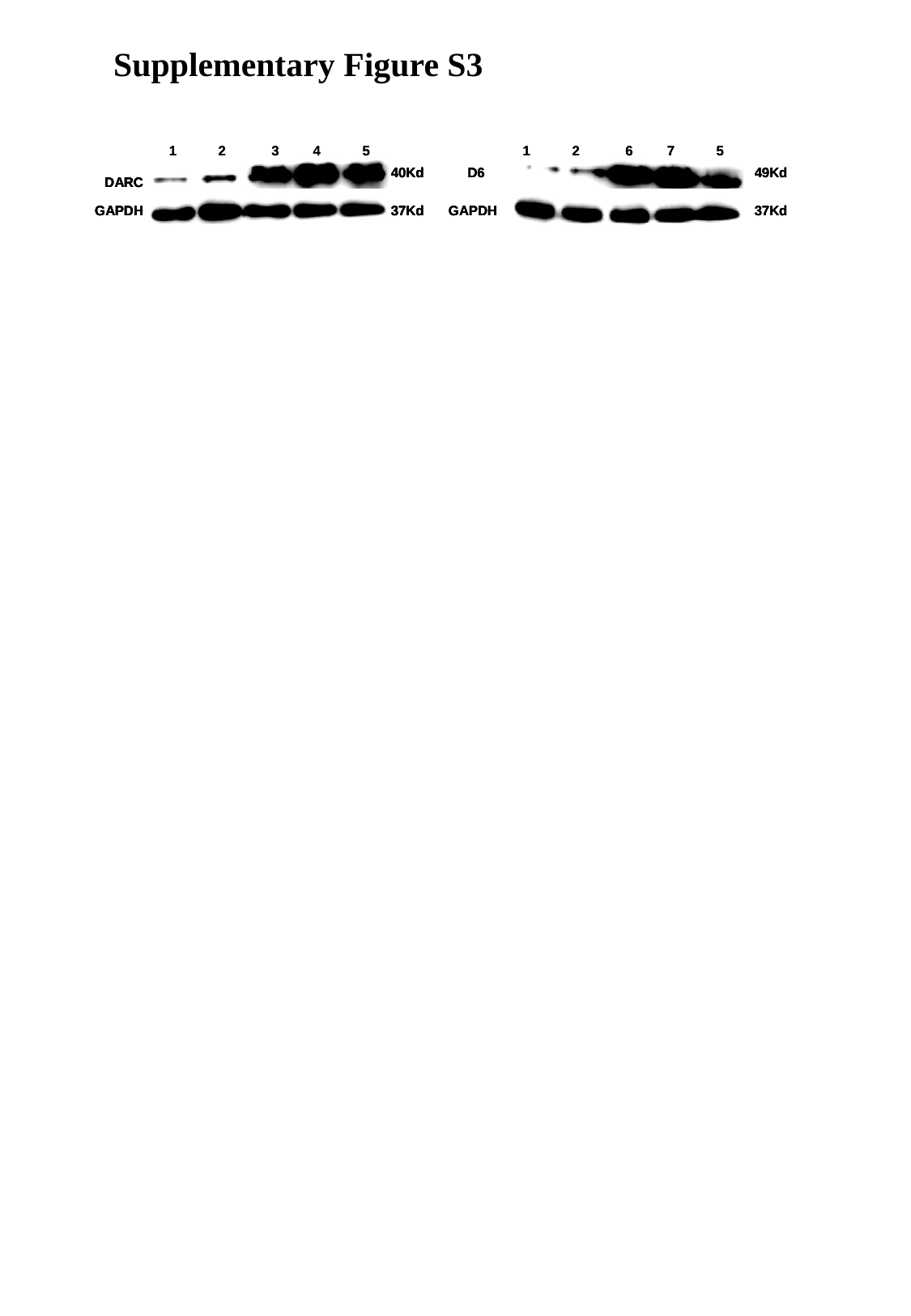

Supplementary Figure S3

Supplement: Figure S3 — DARC and D6 expression levels in xenograft tumors. Representative pictures of DARC and D6 expression levels in xenografts assessed by western blot: lane 1, MDA-MB-231; lane 2, 231-vect; lane 3, 231-DARC-42G; lane 4, 231-DARC-42D; lane 5, 231-DARC-42D-D6-373Y; lane 6, 231-D6-373S; lane 7, 231-D6-373Y. (PPT) [file pone.0078901.s004.ppt]
