# Supplementary material for: Effect of Genetic Variants in Two Chemokine Decoy Receptor Genes, DARC and CCBP2, on Metastatic Potential of Breast Cancer
Source: PLoS One. 2013 Nov 15;8(11):e78901. doi: 10.1371/journal.pone.0078901 (PMC3829817; doi:10.1371/journal.pone.0078901)
Supplement: Table S1 — Primers for the plasmid constructs, RT-PCR and real-time PCR. (DOC) [file pone.0078901.s005.doc]

**Table S1**

**Primers for the plasmid constructs, RT-PCR and real-time PCR**

| Gene | Primers | | Product | Tm |
| --- | --- | --- | --- | --- |
| **Primers for plasmid constructs** | | | | |
| *DARC* | Up | 5’-GGGGTACCCCAccATGGGGAACTGTCTGC-3’ | 1025 bp | 60° |
|  | Down | 5’-GCTCTAGAGCTGGGAAGAGAACTAGGATTTG-3’ |  |  |
| *CCBP2* | Up | 5’- GGGGTACCCCaacAtggccgccactg-3’ | 1170 bp | 60° |
|  | Down | 5’-CGGAATTCCGaaatttggtcacTcaggctg-3’ |  |  |
| **Primers for RT-PCR** | | | | |
| *DARC* | Up | 5’- CCTGTGGGCCTGGTTTATTTTCT-3’ | 303 bp | 60° |
|  | Down | 5’- ATTCAGGTTGACAGGTGGGAAGA-3’ |  |  |
| *CCBP2* | Up | 5’-CCTGCTCCTTGCTACCATAGTATGG-3’ | 232 bp | 60° |
|  | Down | 5’-CACCAAGACACAACCAATACGGGAG-3’ |  |  |
| *GAPDH* | Up | 5’- GGGAGC CAAAAGGGTCATCATCTC-3’ | 353 bp | 60° |
|  | Down | 5’- CCATGCCAGT GAGCTTCCCGTTC-3’ |  |  |
| **Primers for real-time PCR** | | | | |
| *DARC* | Up | 5’-CCCTCAACTGAGAACTCAAGTC -3’ | 233 bp | 60° |
|  | Down | 5’-CGGAAGAGAGGTCTGAAAAGCA -3’ |  |  |
| *CCBP2* | Up | 5’-CTGAGGATGCCGATTCTGAGA -3’ | 187 bp | 60° |
|  | Down | 5’-TAACGGAGCAAGACCATGAGA -3’ |  |  |
| *CDKN1A* | Up | 5’-CTGCCCAAGCTCTACCTTCC-3’ | 265 bp | 60° |
|  | Down | 5’-CCCGCAGTATCTTGCCTCC-3’ |  |  |
| *TBX2* | Up | 5’-CATCTGCGCTCCCTCAAGAG-3’ | 108 bp | 60° |
|  | Down | 5’-CGTGCCTAGCTTGTGGAACT-3’ |  |  |
| *CDKN1B* | Up | 5’-ATCACAAACCCCTAGAGGGCA-3’ | 169 bp | 58° |
|  | Down | 5’-GGAGCCCCAATTAAAGGCG-3’ |  |  |
| *BMI1* | Up | 5’-CCACCTGATGTGTGTGCTTTG-3’ | 162 bp | 58° |
|  | Down | 5’-TTCAGTAGTGGTCTGGTCTTGT-3’ |  |  |
| *RB1* | Up | 5’-GCCTCTCGTCAGGCTTGAG-3’ | 210 bp | 60° |
|  | Down | 5’-TCATCTAGGTCAACTCGTGCAA-3’ |  |  |
| *GAPDH* | Up | 5’-ATGGGGAAGGTGAAGGTCG-3’ | 108 bp | 60° |
|  | Down | 5’-GGGGTCATTGATGGCAACAATA-3’ |  |  |
