# Supplementary material for: Effect of Genetic Variants in Two Chemokine Decoy Receptor Genes, DARC and CCBP2, on Metastatic Potential of Breast Cancer
Source: PLoS One. 2013 Nov 15;8(11):e78901. doi: 10.1371/journal.pone.0078901 (PMC3829817; doi:10.1371/journal.pone.0078901)
Supplement: Table S2 — Primers for discovering SNPs in DARC . (DOC) [file pone.0078901.s006.doc]

**Table S2**

**Primers for discovering SNPs in *DARC***

| Primers | | Product | Tm |
| --- | --- | --- | --- |
| Up | 5’-TCACCGCTCCCAAAGTCC-3’ | 473 bp | 55° |
| Down | 5’-GGCTGTCGAGGCTGCATAA-3’ |  |  |
| Up | 5’-AGCCTACTTTAACTTGTCAGACCATG-3’ | 438 bp | 56° |
| Down | 5’-ATAAGGGAGGTGCTGGGAAAT-3’ |  |  |
| Up | 5’-GGGGATGGAGGAGCAGTGAG-3’ | 579 bp | 60° |
| Down | 5’-GGCAAACAGCACGGGAAATG-3’ |  |  |
| Up | 5’-CCCCAGAGTCCCTTATCCC-3’ | 624 bp | 55° |
| Down | 5’-GCAGAGTCATCCAGCAGGTTA-3’ |  |  |
| Up | 5’-TGTGAATGATTCCTTCCCAGAT-3’ | 579 bp | 55° |
| Down | 5’-CCCAATGGCAACAAGACAA-3’ |  |  |
| Up | 5’-CGGAGCTGAAGGCTTTGC-3’ | 640 bp | 56° |
| Down | 5’-TTGAGATGGCCGTGACCC-3’ |  |  |
