# Supplementary material for: Effect of Genetic Variants in Two Chemokine Decoy Receptor Genes, DARC and CCBP2, on Metastatic Potential of Breast Cancer
Source: PLoS One. 2013 Nov 15;8(11):e78901. doi: 10.1371/journal.pone.0078901 (PMC3829817; doi:10.1371/journal.pone.0078901)
Supplement: Table S3 — Primers and probes for the SNP Stream platform. (DOC) [file pone.0078901.s007.doc]

**Table S3**

Primers and probes for the SNP Stream platform

| SNP | Primers and Probes | |
| --- | --- | --- |
| *DARC* |  |  |
| rs3027012 | Up | TAGAGATAGCTAGACACACCCAGAC |
|  | Down | TGTGCACGTGGGCGTGAG |
|  | Probe | AGAGCGAGTGACGCATACTATATGTGCACAATGATACACAGCAAA |
| rs2814788 | Up | GCAGACAGAAGGGCTGGG |
|  | Down | TCCCTGCCCAGAACCTGA |
|  | Probe | AGCGATCTGCGAGACCGTATGGCTRTCAGCGCCTGTGCTTCCAAG |
| rs12075 | Up | TGGACTTCGAAGATGTATGGA |
|  | Down | AGAGTCATCCAGCAGGTTACAG |
|  | Probe | CGACTGTAGGTGCGTAACTCGATTCCTTCCCAGATGGAGACTATG |
| *CCBP2* |  |  |
| rs4682857 | Up | ACAAGCCTGCACTTTCTCC |
|  | Down | TCATTCAGTTCTCTGCTCAGAT |
|  | Probe | GTGATTCTGTACGTGTCGCCTCCATCCCATTCACCAAACATGAAA |
| rs4682859 | Up | AATAAAATATGGAAAGATATACTCCTCA |
|  | Down | AACTTAAAAGGATGTGAAAGGTTTTA |
|  | Probe | CGTGCCGCTCGTGATAGAATATAGTAATGGTGGCTTTATCTGAAC |
| rs4683342 | Up | CCCTCCTTCCTCCTTCTG |
|  | Down | CTAATGCACCAATCATCTCTTCTA |
|  | Probe | CGACTGTAGGTGCGTAACTCTTACTATGGGGTACAGTTGGGTGAA |
| rs9815043 | Up | TGATCCACCCACCTTGGC |
|  | Down | TTAGCCTCTGTTCTGCTCTACC |
|  | Probe | AGATAGAGTCGATGCCAGCTGCTGGAATTACAAGCATGAGCCACC |
| rs3732859 | Up | ATGAAAATCCCAAGGGTGT |
|  | Down | TAGGAGGTTCTGCTGGAAGC |
|  | Probe | GTGATTCTGTACGTGTCGCCGGAGGAAGAGCTTCCAAATGGTCCC |
| rs2228468 | Up | TTCCCACCAGACCAAAATT |
|  | Down | ATTATCCAGCTGTTCTGAGAGC |
|  | Probe | GGCTATGATTCGCAATGCTTATTCCCCACATCCTCCTTGTTAGGG |
| rs1366046 | Up | AAACATGACAGTTTCAGTTGGC |
|  | Down | ATTTCAAAGTTACTTTCTATATGCGG |
|  | Probe | GGATGGCGTTCCGTCCTATTATAATGCAGACAGTAACCATGCTCA |
